# Crystal structure and Hirshfeld surface analysis of (*E*)-4-{[2-(4-hy­droxy­benzo­yl)hydrazin-1-yl­idene]meth­yl}pyridin-1-ium nitrate

**DOI:** 10.1107/S2056989018002141

**Published:** 2018-02-07

**Authors:** Mir Abolfazl Naziri, Ertan Şahin, Tuncer Hökelek

**Affiliations:** a Department of Inorganic Chemistry, Atatürk University, Erzurum, Turkey; b Department of Physics, Hacettepe University, 06800 Beytepe, Ankara, Turkey

**Keywords:** crystal structure, aroyl hydrazone, Schiff base, hydrogen bonding, π–π inter­actions, Hirshfeld surface

## Abstract

The asymmetric unit of the title aroyl hydrazone Schiff base salt, C_13_H_12_N_3_O_2_^+^·N O_3_^−^, consists of one mol­ecular cation in the keto tautomeric form, adopting an *E* configuration with respect to the azomethine bond, and one nitrate anion. The two units are linked *via* an N—H⋯O hydrogen bond. The mol­ecule overall is non-planar, with the pyridinium and benzene rings being inclined to each other by 4.21 (4)°. In the crystal, cations and anions are linked *via* inter­molecular O—H⋯O and bifurcated N—H⋯O hydrogen bonds, forming a two-dimensional network parallel to (101). These networks are further linked by C—H⋯O hydrogen bonds, forming slabs parallel to (101). The slabs are linked by offset π–π inter­actions, involving the benzene and pyridinium rings of adjacent slabs [inter­centroid distance = 3.610 (2) Å], forming a three-dimensional structure. The Hirshfeld surface analysis of the crystal structure indicates that the most important contributions for the crystal packing are from H⋯O/O⋯H (45.1%), H⋯H (19.3%), H⋯C/C⋯H (14.5%), H⋯N/N⋯H (7.9%) and C⋯C (6.0%) inter­actions.

## Abstract 2

The title aroyl hydrazone Schiff base salt, consists of one mol­ecular cation in the keto tautomeric form, adopting an *E* configuration with respect to the azomethine bond, and one nitrate anion.

## Chemical context   

Hydrazone Schiff bases and their coordination compounds have gained importance recently because of their application as models in biological, analytical and anti­microbial systems, and also due to their anti­cancer, anti­bacterial as well as anti­fungal activities (Ruben *et al.*, 2003 ▸). Aroyl hydrazones are a class of versatile ligands, capable of generating various mol­ecular architectures and coordination polyhedra (Ruben *et al.*, 2003 ▸; Uppadine Gisselbrecht & Lehn, 2004 ▸; Uppadine & Lehn, 2004 ▸; Wood *et al.*, 2004 ▸). Aroyl hydrazones are obtainable through hydrazide-ketone/aldehyde condensation, and they exhibit flexible metal-chelating capabilities through their keto–enol tautomerism and possible reversible deprotonation. The empty N,O-donor chelating pockets of aroyl hydrazones that are incorporated into frameworks can potentially make them amenable to post-synthetic metalation (Evans *et al.*, 2014 ▸). The structure determination of the title aroyl hydrazone Schiff base salt was undertaken in order to compare the results obtained with those reported previously. In this context, we synthesized the title compound, (*E*)-4-{[2-(4-hy­droxy­benzo­yl)hydrazin-1-yl­idene]meth­yl}pyridin-1-ium nitrate, and report herein on its crystal and mol­ecular structures along with the Hirshfeld surface analysis.
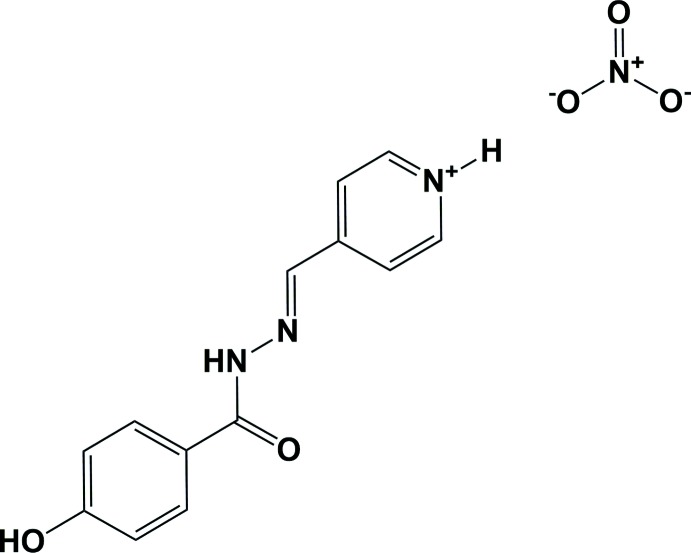


## Structural commentary   

**Figure 1:**
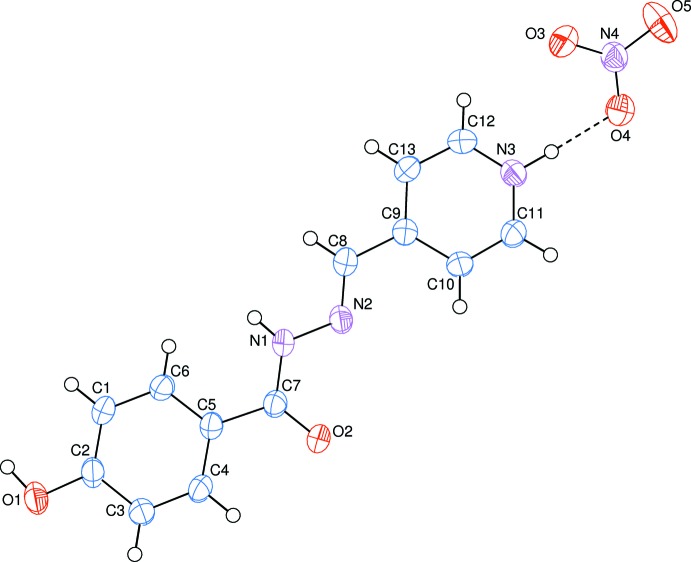
The mol­ecular structure of the title aroyl hydrazone Schiff base salt, with the atom-numbering scheme. The N—H_Pym_⋯O_N_ (Pym = pyridinium and N = nitrate) hydrogen bond (see Table 1 ▸) is shown as a dashed line. Displacement ellipsoids are drawn at the 50% probability level.

The asymmetric unit of the title aroyl hydrazone Schiff base salt contains one mol­ecular cation and one nitrate anion, which are linked *via* an N^+^—H ⋯ O hydrogen bond (Fig. 1 ▸, Table 1 ▸). The organic cation is in the keto tautomeric form, which can be verified from the C=O and C—NH bond lengths of the amide unit: O2=C7 is 1.228 (2) Å and N1—C7 is 1.359 (2) Å. Amide unit bond lengths for aroyl hydrazones are in the ranges 1.218–1.292 Å for C=O bonds and 1.313–1.365 Å for C–N bonds in the keto tautomeric form, and 1.284–1.314 Å for C=O bonds and 1.291–1.331 Å for C—N bonds in the enol tautomeric form (Hosseini-Monfared *et al.*, 2013 ▸). The three bond angles around atom C7, *viz*. O2—C7—N1 [121.65 (15)°], O2—C7—C5 [122.00 (15)°] and N1—C7—C5 [116.35 (14)°], differ from 120°, probably in order to decrease the repulsion between the lone pairs present on atoms N1 and O2.

The configuration at the N2=C8 [1.276 (2) Å] bond is *E*, where torsion angle N1—N2—C8—C9 is −177.58 (14)°. On the other hand, torsion angles N2—N1—C7—C5 and C8—N2—N1—C7 are −179.66 (13) and −178.09 (15)°, respectively, and the benzene (C1–C6) and pyridinium (N3/C9–C13) rings are oriented at a dihedral angle of 4.21 (4)°, probably due to the steric inter­actions between the hydrogen atoms (Table 2 ▸). Thus, the mol­ecule is non-planar as a whole. The central C9—C8=N2—N1—C7=O2 moiety of the mol­ecular cation adopts an extended double-bonded conformation and has a maximum deviation of 0.0331 (18) Å for atom C8, from the mean plane.

## Supra­molecular features   

**Figure 2:**
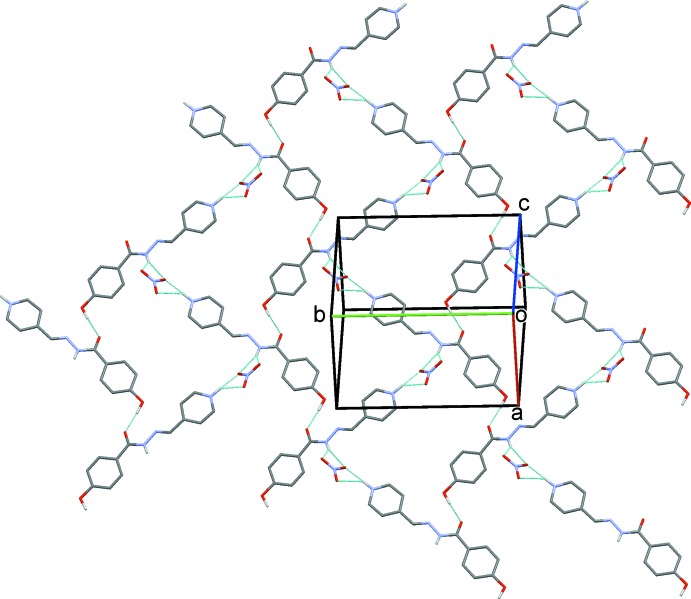
Part of the crystal structure, viewed normal to (101). The O—H⋯O and N—H⋯O hydrogen bonds (see Table 1 ▸) are shown as dashed lines, and C-bound H atoms have been omitted for clarity.

**Figure 3:**
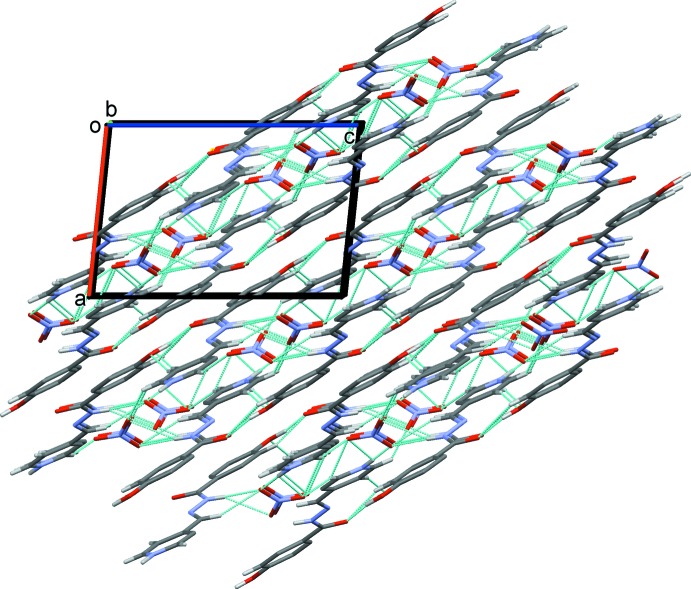
A view along the *b* axis of the crystal packing of the title compound. The hydrogen bonds are shown as dashed lines (see Table 1 ▸), and only H atoms involved in these inter­actions have been included.

Hydrogen bonding and van der Waals contacts are the dominant inter­actions in the crystal packing. In the crystal, O—H_Hydr_⋯O_Hydrz_, N—H_Pym_⋯O_N_ and bifurcated N—H_Hydrz_⋯O_N_ (Hydr = hy­droxy, Hydrz = hydrazide, Pym = pyridinium and N = nitrate) hydrogen bonds (Table 1 ▸) link the cations and anions into a two-dimensional network parallel to (101), as illustrated in Fig. 2 ▸. A series of C—H⋯O hydrogen bonds [C—H_Bnz_⋯O_Hydrz_, C—H_Pym_⋯O_N_, C—H_Pym_⋯O_Hydr_ and C—H_Meth_⋯O_N_ (Bnz = benzene and Meth = methine)] link adjacent layers, forming slabs parallel to (101); see Fig. 3 ▸. The slabs are linked by offset π–π inter­actions, forming a three-dimensional structure. The offset π–π inter­action between the benzene (*Cg*1 is the centroid of atoms C1–C6) and pyridinium (*Cg*2 is the centroid of atoms N3/C9–C13) rings of adjacent slabs has an inter­centroid *Cg*1⋯*Cg*2(−*x* + 2, −*y* + 1, −*z* + 1) distance of 3.610 (2) Å, while α is 4.2 (1)°, and the inter­planar distances are 3.263 (7) and 3.366 (7) Å, with an offset distance of 1.303 Å.

## Hirshfeld surface analysis   

**Figure 4:**
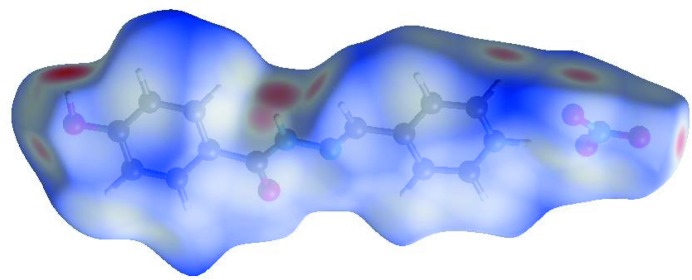
View of the three-dimensional Hirshfeld surface of the title aroyl hydrazone Schiff base salt plotted over *d*_norm_ in the range −0.6521 to 1.7041 a.u.

**Figure 5:**
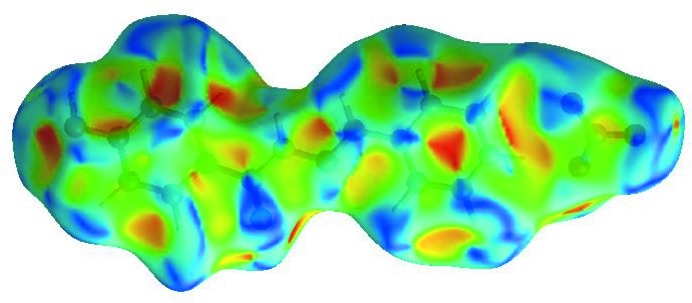
Hirshfeld surface of the title aroyl hydrazone Schiff base salt plotted over shape-index.

**Figure 6:**
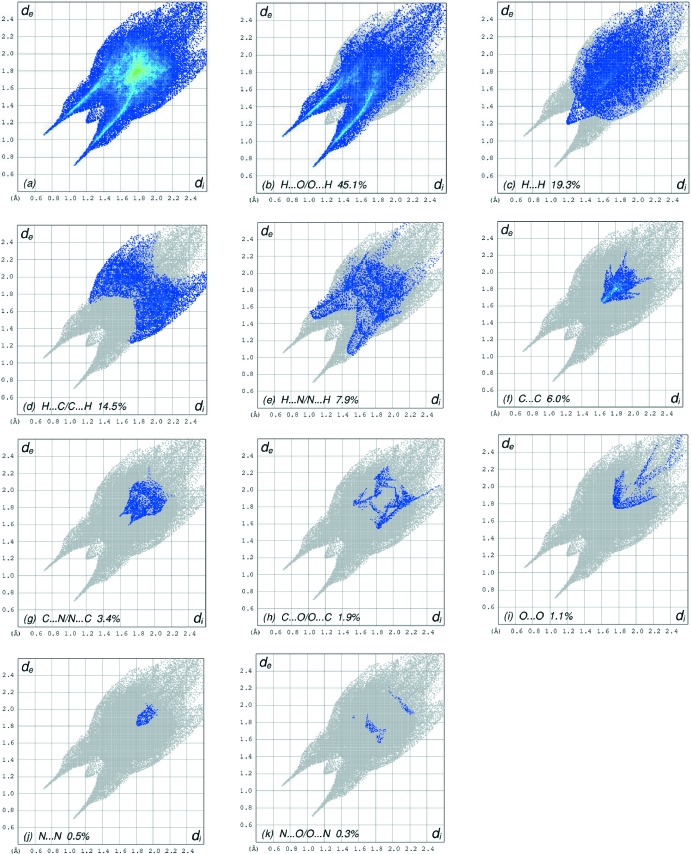
The full two-dimensional fingerprint plots for the title aroyl hydrazone Schiff base salt, showing (*a*) all inter­actions, and delineated into (*b*) H⋯O/O⋯H, (*c*) H⋯H, (*d*) H⋯C/C⋯H, (*e*) H⋯N/N⋯H, (*f*) C⋯C, (*g*) C⋯N/N⋯C, (*h*) C⋯O/O⋯C, (*i*) O⋯O, (*j*) N⋯N and (*k*) N⋯O/O⋯N inter­actions. The *d*_i_ and *d*_e_ values are the closest inter­nal and external distances (in Å) from given points on the Hirshfeld surface contacts.

In order to visualize the inter­molecular inter­actions in the crystal of the title aroyl hydrazone Schiff base salt, a Hirshfeld surface (HS) analysis (Hirshfeld, 1977 ▸; Spackman & Jayatilaka, 2009 ▸) was carried out by using *Crystal Explorer 17.5* (Turner *et al.*, 2017 ▸). In the HS plotted over *d*_norm_ (Fig. 4 ▸), the white surfaces indicate contacts with distances equal to the sum of van der Waals radii, and the red and blue colours indicate distances shorter (in close contact) or longer (distinct contact) than the van der Waals radii, respectively (Venkatesan *et al.*, 2016 ▸). The bright-red spots appearing near N—O4, N—O5 and hydrogen atoms H1*A*, H1*B* and H3*A* indicate their role as the respective donors and acceptors in the dominant O—H⋯O and N—H⋯O hydrogen bonds (Spackman *et al.*, 2008 ▸; Jayatilaka *et al.*, 2005 ▸). The shape-index of the HS is a tool to visualize the π–π stacking by the presence of adjacent red and blue triangles; if there are no adjacent red and/or blue triangles, then there are no π–π inter­actions. Fig. 5 ▸ clearly suggest that there are π–π inter­actions in (I). The overall two-dimensional fingerprint plot and those delineated into H⋯O/O⋯H, H⋯H, H⋯C/C⋯H, H⋯N/N⋯H, C⋯C, C⋯N/N⋯C, C⋯O/O⋯C, O⋯O, N⋯N and N⋯O/O⋯N contacts (McKinnon *et al.*, 2007 ▸) are illustrated in Fig. 5 ▸ *a*–*k*, respectively, together with their relative contributions to the Hirshfeld surface. The most important inter­action is H⋯O/O⋯H contributing 45.1% to the overall crystal packing, which is reflected in Fig. 6 ▸ *b* as pair of spikes with the tips at *d*_e_ + *d*_i_ ∼1.75 Å. The short H⋯O/O⋯H contacts are masked by strong O—H⋯O hydrogen bonding in this plot. In the fingerprint plot delineated into H⋯H contacts (Fig. 6 ▸ *c*), the 19.3% contribution to the overall crystal packing is reflected as widely scattered points of high density due to the large hydrogen content of the mol­ecule. The single spike in the centre at *d*_e_ = *d*_i_ = 1.2 Å in Fig. 5 ▸ *c* is due to the short inter­atomic H ⋯ H contacts (Table 2 ▸). In the absence of C—H⋯π inter­actions in the crystal, the pair of characteristic wings resulting in the fingerprint plot delineated into H⋯C/C⋯H contacts with 14.5% contribution to the HS, Fig. 6 ▸ *d*, and the pair of thin edges at *d*_e_ + *d*_i_ ∼1.93 Å result from short inter­atomic H⋯C/C⋯H contacts (Table 2 ▸). The H⋯N/N⋯H contacts in the structure with 7.9% contribution to the HS has a symmetrical distribution of points, Fig. 5 ▸ *e*, with the tips at *d*_e_ + *d*_i_ ∼1.52 Å arising from the short inter­atomic H⋯N/N⋯H contacts listed in Table 2 ▸. The C⋯C contacts assigned to short inter­atomic C⋯C contacts with 6.0% contribution to the HS appear as an arrow-shaped distribution of points in Fig. 6 ▸ *f*, with the vertex at *d*_e_ = *d*_i_ ∼1.65 Å. Finally, the C⋯N/N⋯C (Fig. 6 ▸ *g*) and C⋯O/O⋯C (Fig. 6 ▸ *h*) contacts in the structure with 3.4% and 1.9% contributions to the HS have nearly symmetrical distributions of points, with the scattered points of low densities.

**Figure 7:**
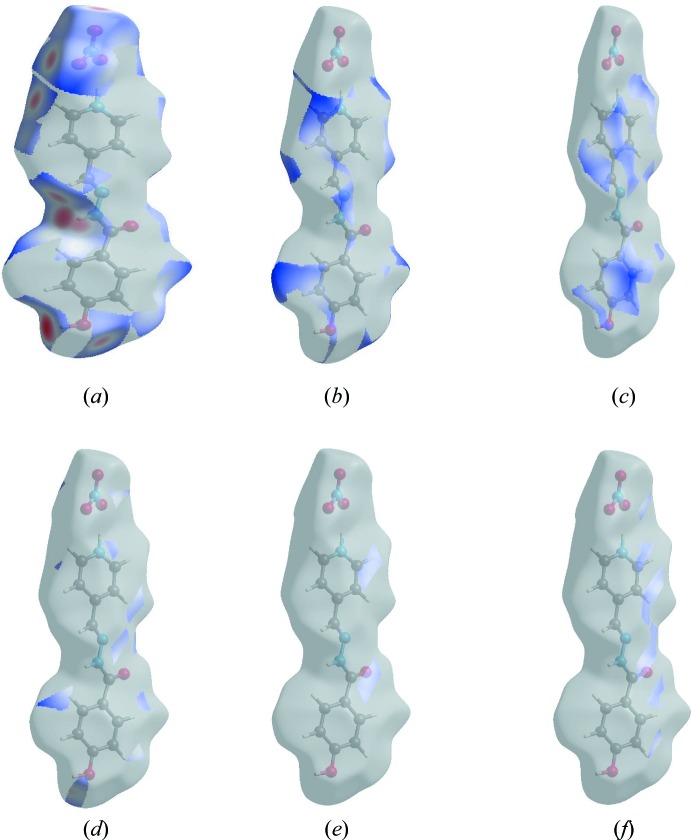
The Hirshfeld surface representations with the function *d*_norm_ plotted onto the surface for (*a*) H⋯O/O⋯H, (*b*) H⋯H, (*c*) H⋯C/C⋯H, (*d*) H⋯N/N⋯H, (*e*) C⋯C and (*f*) C⋯N/N⋯C inter­actions.

The Hirshfeld surface representations with the function *d*_norm_ plotted onto the surface are shown for the H⋯O/O⋯H, H⋯H, H⋯C/C⋯H, H⋯N/N⋯H, C⋯C and C⋯N/N⋯C inter­actions in Fig. 7 ▸ *a*–*f*, respectively.

The Hirshfeld surface analysis confirms the importance of H-atom contacts in establishing the packing. The large number of H⋯O/O⋯H, H⋯H and H⋯C/C⋯H inter­actions suggest that van der Waals inter­actions and hydrogen bonding play the major roles in the crystal packing (Hathwar *et al.*, 2015 ▸).

## Synthesis and crystallization   

The title compound was prepared by the reaction of Cd(NO_3_)_2_·4H_2_O (0.15 g, 0.5 mmol) and 4-[(4-hy­droxybenzo­yl)hydrazonemeth­yl]pyridin (0.12 g, 0.5 mmol) in ethanol by using a branched-tube method (Shaabani *et al.*, 2017 ▸). After two months, the formation of yellow-coloured crystals was observed. They were filtered off and washed several times with hot ethanol for purification (yield: 0.20 g, 74%, m.p. 613 K). Analysis calculated for C_13_H_12_N_4_O_5_: C, 51.32; H, 3.98; N, 17.41. Found: C, 51.08; H, 4.14; N, 17.09. Characteristic IR bands (cm^−1^): 3526 *m*, ν(OH); 1375 *m*, ν(N—O); 1644 *s*, ν(C=N); 1501 *s*, ν(N=O); 1105 *s*, ν(NN).

## Refinement   

Crystal data, data collection and structure refinement details are summarized in Table 3 ▸. H atoms of the OH and NH groups were located in a difference-Fourier map and refined freely. The C-bound H atoms were positioned geometrically with C—H = 0.93 Å, and refined as riding with *U*_iso_(H) = 1.2*U*_eq_(C). The highest residual electron density was found 2.48 Å from atom H1.

## Supplementary Material

Crystal structure: contains datablock(s) I, global. DOI: 10.1107/S2056989018002141/su5422sup1.cif

Structure factors: contains datablock(s) I. DOI: 10.1107/S2056989018002141/su5422Isup2.hkl

Supporting information file. DOI: 10.1107/S2056989018002141/su5422Isup3.cml

CCDC reference: 1822116

Additional supporting information:  crystallographic information; 3D view; checkCIF report

## Figures and Tables

**Table 1:** Hydrogen-bond geometry (Å, °)

*D*—H⋯*A*	*D*—H	H⋯*A*	*D*⋯*A*	*D*—H⋯*A*
O1—H1*A*⋯O2^i^	0.96 (2)	1.79 (2)	2.742 (2)	170 (2)
N1—H1*B*⋯O4^iii^	0.84 (2)	2.25 (2)	3.057 (2)	161 (2)
N1—H1*B*⋯O5^iii^	0.84 (2)	2.47 (2)	3.174 (3)	141 (2)
N3—H3*A*⋯O4	0.97 (2)	1.80 (2)	2.763 (2)	178 (2)
C1—H1⋯O2^i^	0.93	2.58	3.258 (2)	130
C8—H8⋯O4^iii^	0.93	2.42	3.243 (2)	148
C10—H10⋯O1^ii^	0.93	2.48	3.375 (2)	162
C11—H11⋯O5^iv^	0.93	2.42	3.104 (3)	130
C12—H12⋯O3^v^	0.93	2.34	3.191 (2)	152

Symmetry codes: (i) 
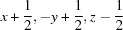
; (ii) 
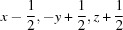
; (iii) 
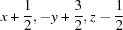
; (iv) 
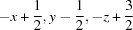
; (v) 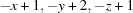.

**Table 2:** Selected interatomic distances (Å)

O1⋯H10^i^	2.48	C7⋯H1*A* ^ii^	2.72 (2)
O2⋯H1^ii^	2.58	H1⋯H1*A*	2.28
O2⋯H1*A* ^ii^	1.79 (2)	H1*B*⋯O4^iii^	2.25 (2)
O2⋯H4	2.52	H1*B*⋯O5^iii^	2.47 (2)
O3⋯H3*A*	2.48 (2)	H1*B*⋯N4^iii^	2.73 (2)
O3⋯H12	2.59	H6⋯O4^iii^	2.71
O4⋯H3*A*	1.80 (2)	H6⋯O5^iii^	2.75
N1⋯H6	2.59	H6⋯N4^iii^	2.81
N2⋯H1*A* ^ii^	2.56 (2)	H6⋯H1*B*	2.11
N2⋯H10	2.62	H8⋯O4^iii^	2.42
N4⋯H3*A*	2.47 (2)	H8⋯H1*B*	2.12
C6⋯H1*B*	2.61 (2)	H8⋯H13	2.46

Symmetry codes: (i) 
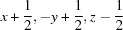
; (ii) 
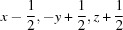
; (iii) 
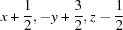
.

**Table 3:** Experimental details

Crystal data	
Chemical formula	C_13_H_12_N_3_O_2_^+^·NO_3_^−^
*M*_r_	304.27
Crystal system, space group	Monoclinic, *P*2_1_/*n*
Temperature (K)	296
*a*, *b*, *c* (Å)	8.335 (3), 13.929 (5), 12.184 (4)
β (°)	95.902 (10)
*V* (Å^3^)	1407.1 (8)
*Z*	4
Radiation type	Mo *K*α
μ (mm^−1^)	0.11
Crystal size (mm)	0.15 × 0.14 × 0.12

Data collection	
Diffractometer	Bruker APEXII CCD
Absorption correction	Multi-scan (*SADABS*; Bruker, 2012 ▸)
*T*_min_, *T*_max_	0.945, 0.976
No. of measured, independent and observed [*I* > 2σ(*I*)] reflections	44410, 3499, 2508
*R*_int_	0.052
(sin θ/λ)_max_ (Å^−1^)	0.669

Refinement	
*R*[*F* ^2^ > 2σ(*F* ^2^)], *wR*(*F* ^2^), *S*	0.049, 0.149, 1.04
No. of reflections	3499
No. of parameters	211
H-atom treatment	H atoms treated by a mixture of independent and constrained refinement
Δρ_max_, Δρ_min_ (e Å^−3^)	0.75, −0.25

Computer programs: *APEX2* and *SAINT* (Bruker, 2012 ▸), *SHELXS97* and *SHELXL97* (Sheldrick, 2008 ▸), *ORTEP-3 for Windows* and *WinGX* (Farrugia, 2012 ▸), *Mercury* (Macrae *et al.*, 2008 ▸) and *PLATON* (Spek, 2015 ▸).

## supplementary crystallographic information

### Crystal data

**Table:**

C_13_H_12_N_3_O_2_^+^·NO_3_^−^	*F*(000) = 632
*M**_r_* = 304.27	*D*_x_ = 1.436 Mg m^−^^3^
Monoclinic, *P*2_1_/*n*	Mo *K*α radiation, λ = 0.71073 Å
Hall symbol: -P 2yn	Cell parameters from 9700 reflections
*a* = 8.335 (3) Å	θ = 3.1–27.5°
*b* = 13.929 (5) Å	µ = 0.11 mm^−^^1^
*c* = 12.184 (4) Å	*T* = 296 K
β = 95.902 (10)°	Block, colourless
*V* = 1407.1 (8) Å^3^	0.15 × 0.14 × 0.12 mm
*Z* = 4	

### Data collection

**Table:**

Bruker APEXII CCD diffractometer	3499 independent reflections
Radiation source: fine-focus sealed tube	2508 reflections with *I* > 2σ(*I*)
Graphite monochromator	*R*_int_ = 0.052
φ and ω scans	θ_max_ = 28.4°, θ_min_ = 2.9°
Absorption correction: multi-scan (*SADABS*; Bruker, 2012)	*h* = −11→10
*T*_min_ = 0.945, *T*_max_ = 0.976	*k* = −18→18
44410 measured reflections	*l* = −16→16

### Refinement

**Table:**

Refinement on *F*^2^	Primary atom site location: structure-invariant direct methods
Least-squares matrix: full	Secondary atom site location: difference Fourier map
*R*[*F*^2^ > 2σ(*F*^2^)] = 0.049	Hydrogen site location: inferred from neighbouring sites
*wR*(*F*^2^) = 0.149	H atoms treated by a mixture of independent and constrained refinement
*S* = 1.04	*w* = 1/[σ^2^(*F*_o_^2^) + (0.0782*P*)^2^ + 0.4149*P*] where *P* = (*F*_o_^2^ + 2*F*_c_^2^)/3
3499 reflections	(Δ/σ)_max_ < 0.001
211 parameters	Δρ_max_ = 0.75 e Å^−^^3^
0 restraints	Δρ_min_ = −0.25 e Å^−^^3^

### Special details

**Table:**

Geometry. All esds (except the esd in the dihedral angle between two l.s. planes) are estimated using the full covariance matrix. The cell esds are taken into account individually in the estimation of esds in distances, angles and torsion angles; correlations between esds in cell parameters are only used when they are defined by crystal symmetry. An approximate (isotropic) treatment of cell esds is used for estimating esds involving l.s. planes.
Refinement. Refinement of F^2^ against ALL reflections. The weighted R-factor wR and goodness of fit S are based on F^2^, conventional R-factors R are based on F, with F set to zero for negative F^2^. The threshold expression of F^2^ > 2sigma(F^2^) is used only for calculating R-factors(gt) etc. and is not relevant to the choice of reflections for refinement. R-factors based on F^2^ are statistically about twice as large as those based on F, and R- factors based on ALL data will be even larger.

### Fractional atomic coordinates and isotropic or equivalent isotropic displacement parameters (Å^2^)

**Table:**

	*x*	*y*	*z*	*U*_iso_*/*U*_eq_	
O1	1.15687 (19)	0.08055 (9)	0.24127 (12)	0.0576 (4)	
H1A	1.207 (3)	0.1109 (18)	0.183 (2)	0.080 (8)*	
O2	0.82328 (18)	0.32288 (9)	0.59284 (11)	0.0549 (4)	
O3	0.3316 (2)	0.99298 (10)	0.57667 (11)	0.0619 (4)	
O4	0.37385 (19)	0.95570 (10)	0.74819 (11)	0.0595 (4)	
O5	0.22205 (19)	1.07556 (11)	0.69841 (15)	0.0730 (5)	
N1	0.81569 (17)	0.43407 (10)	0.45738 (12)	0.0392 (3)	
H1B	0.830 (3)	0.4512 (15)	0.3928 (19)	0.055 (6)*	
N2	0.74051 (16)	0.49881 (10)	0.51883 (11)	0.0368 (3)	
N3	0.50122 (17)	0.80177 (10)	0.64341 (12)	0.0408 (3)	
H3A	0.456 (3)	0.8548 (17)	0.6811 (19)	0.069 (7)*	
N4	0.30832 (18)	1.00915 (10)	0.67253 (13)	0.0438 (4)	
C1	1.08209 (19)	0.24460 (12)	0.27240 (13)	0.0367 (4)	
H1	1.1286	0.2655	0.2104	0.044*	
C2	1.0865 (2)	0.14803 (12)	0.30031 (14)	0.0386 (4)	
C3	1.0173 (2)	0.11743 (13)	0.39341 (15)	0.0452 (4)	
H3	1.0208	0.0528	0.4129	0.054*	
C4	0.9439 (2)	0.18265 (12)	0.45671 (14)	0.0410 (4)	
H4	0.8980	0.1616	0.5189	0.049*	
C5	0.93716 (18)	0.27990 (11)	0.42900 (13)	0.0334 (3)	
C6	1.00883 (18)	0.30970 (12)	0.33645 (13)	0.0353 (4)	
H6	1.0073	0.3744	0.3176	0.042*	
C7	0.85500 (18)	0.34613 (11)	0.50025 (13)	0.0352 (3)	
C8	0.7030 (2)	0.57893 (12)	0.47233 (14)	0.0417 (4)	
H8	0.7236	0.5893	0.3997	0.050*	
C9	0.62801 (18)	0.65447 (11)	0.53250 (13)	0.0347 (3)	
C10	0.56331 (19)	0.63741 (12)	0.63168 (14)	0.0377 (4)	
H10	0.5629	0.5758	0.6610	0.045*	
C11	0.5004 (2)	0.71264 (12)	0.68521 (15)	0.0402 (4)	
H11	0.4565	0.7019	0.7513	0.048*	
C12	0.5620 (2)	0.82024 (12)	0.54903 (15)	0.0441 (4)	
H12	0.5613	0.8827	0.5221	0.053*	
C13	0.6257 (2)	0.74791 (12)	0.49147 (14)	0.0401 (4)	
H13	0.6673	0.7610	0.4251	0.048*	

### Atomic displacement parameters (Å^2^)

**Table:**

	*U*^11^	*U*^22^	*U*^33^	*U*^12^	*U*^13^	*U*^23^
O1	0.0805 (10)	0.0412 (7)	0.0580 (9)	−0.0054 (6)	0.0395 (8)	−0.0132 (6)
O2	0.0797 (10)	0.0457 (7)	0.0451 (7)	0.0120 (6)	0.0345 (7)	0.0058 (6)
O3	0.0884 (11)	0.0562 (9)	0.0452 (8)	0.0093 (7)	0.0264 (7)	0.0067 (6)
O4	0.0837 (10)	0.0566 (8)	0.0401 (7)	0.0142 (7)	0.0156 (7)	−0.0006 (6)
O5	0.0723 (10)	0.0593 (9)	0.0875 (12)	0.0217 (8)	0.0090 (9)	−0.0260 (8)
N1	0.0496 (8)	0.0387 (8)	0.0316 (7)	0.0066 (6)	0.0145 (6)	−0.0011 (6)
N2	0.0385 (7)	0.0374 (7)	0.0360 (7)	0.0026 (5)	0.0107 (6)	−0.0047 (6)
N3	0.0446 (8)	0.0330 (7)	0.0466 (8)	0.0026 (6)	0.0132 (6)	−0.0013 (6)
N4	0.0502 (8)	0.0359 (7)	0.0473 (9)	−0.0017 (6)	0.0142 (7)	−0.0056 (6)
C1	0.0379 (8)	0.0441 (9)	0.0296 (8)	−0.0016 (7)	0.0103 (6)	−0.0001 (7)
C2	0.0416 (8)	0.0398 (9)	0.0364 (8)	−0.0050 (7)	0.0134 (7)	−0.0096 (7)
C3	0.0586 (11)	0.0329 (9)	0.0479 (10)	−0.0051 (7)	0.0236 (8)	−0.0023 (7)
C4	0.0500 (9)	0.0389 (9)	0.0373 (9)	−0.0059 (7)	0.0198 (7)	−0.0004 (7)
C5	0.0327 (7)	0.0369 (8)	0.0315 (8)	−0.0005 (6)	0.0080 (6)	−0.0027 (6)
C6	0.0355 (8)	0.0375 (8)	0.0340 (8)	0.0018 (6)	0.0085 (6)	0.0034 (6)
C7	0.0359 (8)	0.0368 (8)	0.0343 (8)	−0.0011 (6)	0.0104 (6)	−0.0018 (6)
C8	0.0496 (9)	0.0437 (9)	0.0333 (8)	0.0058 (8)	0.0110 (7)	−0.0005 (7)
C9	0.0346 (8)	0.0363 (8)	0.0334 (8)	0.0004 (6)	0.0046 (6)	−0.0009 (6)
C10	0.0421 (8)	0.0314 (8)	0.0412 (9)	−0.0028 (6)	0.0113 (7)	0.0022 (7)
C11	0.0434 (9)	0.0373 (9)	0.0422 (9)	−0.0030 (7)	0.0154 (7)	0.0006 (7)
C12	0.0522 (10)	0.0350 (9)	0.0464 (10)	0.0042 (7)	0.0106 (8)	0.0088 (7)
C13	0.0449 (9)	0.0425 (9)	0.0342 (8)	0.0030 (7)	0.0096 (7)	0.0070 (7)

### Geometric parameters (Å, º)

**Table:**

O1—C2	1.354 (2)	C4—C3	1.376 (2)
O1—H1A	0.96 (3)	C4—H4	0.9300
O2—C7	1.228 (2)	C5—C4	1.396 (2)
O3—N4	1.224 (2)	C5—C6	1.393 (2)
O4—N4	1.264 (2)	C5—C7	1.482 (2)
N1—C7	1.359 (2)	C6—C1	1.379 (2)
N1—H1B	0.84 (2)	C6—H6	0.9300
N2—N1	1.3649 (19)	C8—H8	0.9300
N2—C8	1.276 (2)	C9—C8	1.459 (2)
N3—C11	1.342 (2)	C9—C10	1.393 (2)
N3—C12	1.329 (2)	C9—C13	1.394 (2)
N3—H3A	0.97 (2)	C10—C11	1.367 (2)
N4—O5	1.2325 (19)	C10—H10	0.9300
C1—H1	0.9300	C11—H11	0.9300
C2—C1	1.387 (2)	C12—H12	0.9300
C2—C3	1.391 (2)	C13—C12	1.366 (2)
C3—H3	0.9300	C13—H13	0.9300
			
O1···H10^i^	2.48	C7···H1A^ii^	2.72 (2)
O2···H1^ii^	2.58	H1···H1A	2.28
O2···H1A^ii^	1.79 (2)	H1B···O4^iii^	2.25 (2)
O2···H4	2.52	H1B···O5^iii^	2.47 (2)
O3···H3A	2.48 (2)	H1B···N4^iii^	2.73 (2)
O3···H12	2.59	H6···O4^iii^	2.71
O4···H3A	1.80 (2)	H6···O5^iii^	2.75
N1···H6	2.59	H6···N4^iii^	2.81
N2···H1A^ii^	2.56 (2)	H6···H1B	2.11
N2···H10	2.62	H8···O4^iii^	2.42
N4···H3A	2.47 (2)	H8···H1B	2.12
C6···H1B	2.61 (2)	H8···H13	2.46
			
C2—O1—H1A	109.6 (15)	C6—C5—C7	123.46 (15)
N2—N1—H1B	116.3 (15)	C1—C6—C5	120.92 (15)
C7—N1—N2	119.41 (14)	C1—C6—H6	119.5
C7—N1—H1B	124.2 (15)	C5—C6—H6	119.5
C8—N2—N1	116.08 (14)	O2—C7—N1	121.65 (15)
C11—N3—H3A	120.6 (14)	O2—C7—C5	122.00 (15)
C12—N3—C11	121.66 (15)	N1—C7—C5	116.35 (14)
C12—N3—H3A	117.7 (14)	N2—C8—C9	120.38 (15)
O3—N4—O4	119.23 (15)	N2—C8—H8	119.8
O3—N4—O5	122.40 (17)	C9—C8—H8	119.8
O5—N4—O4	118.37 (16)	C10—C9—C8	122.46 (15)
C2—C1—H1	120.0	C10—C9—C13	118.54 (14)
C6—C1—C2	120.06 (14)	C13—C9—C8	118.96 (15)
C6—C1—H1	120.0	C9—C10—H10	120.5
O1—C2—C1	123.03 (15)	C11—C10—C9	119.00 (15)
O1—C2—C3	117.33 (16)	C11—C10—H10	120.5
C1—C2—C3	119.64 (15)	N3—C11—C10	120.71 (16)
C2—C3—H3	120.0	N3—C11—H11	119.6
C4—C3—C2	120.02 (16)	C10—C11—H11	119.6
C4—C3—H3	120.0	N3—C12—C13	120.30 (16)
C3—C4—C5	120.96 (15)	N3—C12—H12	119.9
C3—C4—H4	119.5	C13—C12—H12	119.9
C5—C4—H4	119.5	C9—C13—H13	120.1
C4—C5—C7	118.16 (14)	C12—C13—C9	119.79 (15)
C6—C5—C4	118.38 (14)	C12—C13—H13	120.1
			
N2—N1—C7—O2	0.7 (2)	C7—C5—C6—C1	−179.26 (15)
N2—N1—C7—C5	−179.66 (13)	C4—C5—C7—O2	15.3 (2)
C8—N2—N1—C7	−178.09 (15)	C4—C5—C7—N1	−164.34 (15)
N1—N2—C8—C9	−177.58 (14)	C6—C5—C7—O2	−164.23 (16)
C12—N3—C11—C10	0.3 (3)	C6—C5—C7—N1	16.1 (2)
C11—N3—C12—C13	0.1 (3)	C5—C6—C1—C2	−0.6 (2)
O1—C2—C1—C6	−179.76 (15)	C10—C9—C8—N2	−13.9 (3)
C3—C2—C1—C6	−0.3 (3)	C13—C9—C8—N2	163.87 (16)
O1—C2—C3—C4	−179.94 (17)	C8—C9—C10—C11	177.61 (16)
C1—C2—C3—C4	0.6 (3)	C13—C9—C10—C11	−0.1 (2)
C5—C4—C3—C2	0.0 (3)	C8—C9—C13—C12	−177.27 (16)
C6—C5—C4—C3	−0.9 (3)	C10—C9—C13—C12	0.6 (2)
C7—C5—C4—C3	179.50 (16)	C9—C10—C11—N3	−0.3 (3)
C4—C5—C6—C1	1.2 (2)	C9—C13—C12—N3	−0.6 (3)

Symmetry codes: (i) *x*+1/2, −*y*+1/2, *z*−1/2; (ii) *x*−1/2, −*y*+1/2, *z*+1/2; (iii) *x*+1/2, −*y*+3/2, *z*−1/2.

### Hydrogen-bond geometry (Å, º)

**Table:**

*D*—H···*A*	*D*—H	H···*A*	*D*···*A*	*D*—H···*A*
O1—H1*A*···O2^i^	0.96 (2)	1.79 (2)	2.742 (2)	170 (2)
N1—H1*B*···O4^iii^	0.84 (2)	2.25 (2)	3.057 (2)	161 (2)
N1—H1*B*···O5^iii^	0.84 (2)	2.47 (2)	3.174 (3)	141 (2)
N3—H3*A*···O4	0.97 (2)	1.80 (2)	2.763 (2)	178 (2)
C1—H1···O2^i^	0.93	2.58	3.258 (2)	130
C8—H8···O4^iii^	0.93	2.42	3.243 (2)	148
C10—H10···O1^ii^	0.93	2.48	3.375 (2)	162
C11—H11···O5^iv^	0.93	2.42	3.104 (3)	130
C12—H12···O3^v^	0.93	2.34	3.191 (2)	152

Symmetry codes: (i) *x*+1/2, −*y*+1/2, *z*−1/2; (ii) *x*−1/2, −*y*+1/2, *z*+1/2; (iii) *x*+1/2, −*y*+3/2, *z*−1/2; (iv) −*x*+1/2, *y*−1/2, −*z*+3/2; (v) −*x*+1, −*y*+2, −*z*+1.
